# A year of JMP online

**DOI:** 10.4103/0971-6203.31142

**Published:** 2007

**Authors:** A. S. Pradhan

**Affiliations:** Editor-in-chief, JMP, c\o, AMPI, RPAD, CTCRS, Mumbai - 400 094, India. E-mail: ambika.s.pradhan@gmail.com

The objectives[1] set while taking a decision to make JMP online were 1- to enable the journal to reach a much wider readership with no bounds and hindrances of postal deliveries, 2- to minimize the delays between the receipts of the manuscripts and their publication and 3- to provide quality publication to attract and encourage authors to choose Journal of Medical Physics (JMP) for the publication of their research work. Although the journey through 2006 has not been very smooth for the editorial board, the approach to achieve the objectives appears to have yielded much better results than expected. It is not practical to examine the effect of each constraint individually; however, this editorial aims at bringing out the highlights of one year's experience and the main features of JMP.

The online publication of the first issue of volume 31 (2006) was delayed a bit (came out on April 10, 2006) but the publication of the subsequent issues (quarterly publication) could catch up and all the four issues planned for the year became available before the end of 2006. This appears to be a record in itself. About the readership of JMP, as per the number of visitors recorded on the site, at the time of writing of this editorial, the journal has been viewed by more than 70000 viewers. This phenomenal growth in the readership not only enabled in achieving the goals set for the journal but also helped considerably in meeting the aims and objectives of the Association of Medical Physicists of India (AMPI) viz, to promote the application of physics to medical and biological sciences, to encourage research and development and education in the field of MEDICAL PHYSICS, to provide a forum for persons engaged or interested in the field of MEDICAL PHYSICS and to disseminate worldwide information in this field. Some of the articles published in JMP have been viewed as much as 1400 times. Till date, the most viewed article of volume 31 has been a review article entitled “*Positron emission tomography - An overview* by *Shukla AK* and *Kumar Utham*” which appeared in issue No. 1 and the least viewed have been the synopses of papers presented at AMPICON-2006 published in issue No. 3. Even the articles of issue No. 4 (with a popular editorial) appears to have attracted more viewers than the synopses of the 127 poster presentations of AMPICON-2006. This may have some bearing on the varying quality of the material and is a point of further retrospection for the editorial board of JMP.

The highlights of the analysis of the performance of the journal during the year 2006 are presented in Table 1. This may reflect on the efficiency of the working of the editorial board. The overall performance appears to be satisfactory and encouraging. The growth in the popularity of the journal may be gauged from the increasing rate of submission of manuscripts. Apart from the articles already published since the first issue of 2006 (including this issue), as on date there are 18 manuscripts in hand under different phases of processing. The analysis of the manuscripts received during 2006 is shown in Table 2. Volume 31 of 2006 had 21 manuscripts (invited/review/original articles and editorials) and synopses of 63 oral and 127 poster papers presented at the Annual AMPI conference. Out of the nine manuscripts not considered for publication, two were withdrawn and seven were rejected. The rejection of three manuscripts was based on the strong recommendations of the referees whereas the rejection of the remaining manuscripts became inevitable because these were considered as casual submissions of stray articles, much beyond the scope of the journal. Every effort was made to provide full opportunity to the authors to improve their manuscripts through the revisions if there existed a possibility of improvement to make it worth publishing through the critical comments of the referees. This approach to minimize the rejection rate may differ from the other journals but the final manuscripts were attempted to be of about the same standard as those published in any other journal.

**Table 1:** Highlights of the analysis of the performance of the journal during the year 2006

*Stages of handling the manuscripts*	*Average of the actual No. of days per manuscript*	*Expected No. of days per manuscript*
Duration between submission and assigning the reviewers by the editor after pre-review	1.7	7
Days until the manuscript is under first review	27.9	21
Time taken by the authors for the first revision	26.0	28
Time taken by the editor for the decision on the revised and re-revised versions and the internal review	5.2	Flexible to the needs
Days from first submission to acceptance	49.5	84
Days from acceptance to publication	74.3	Quarterly publication

**Table 2:** Analysis of manuscripts (excluding synopses of 63 oral and 127 poster papers presented at the Annual AMPI conference) received during 2006 for the acceptance, rejection and under reviewing

*Article type*	*Submitted*	*Accepted*	*Rejected/withdrawn*	*Under review*
Invited/review/original	33	18 (54%)	9 (27%)	6 (18%)
Editorial	5	4 (80%)	1 (20%)	-
Book reviews	1	-	-	1 (100%)
Total	39	22 (56%)	10 (26%)	7 (18%)

JMP continues to share the responsibility within the editorial board members as much as possible. However, the workload could not be shared, as equally as planned, among the 22 members of the board due to the other preoccupations of some of the members. Help of the experts from outside the editorial board in refereeing the manuscripts was found very valuable. In view of this, a reorganization and expansion of the editorial board became inevitable. A need to enlarge the scope was also recognized and features like “NEWS” and “BOOK REVIEW” were considered to be included. A few amendments in the instructions to authors are also made and these may be noted through this issue. The policy of refereeing continues to provide the authors critical comments to improve the manuscripts as much as possible and to minimize the rejection of good work for the lack of the art of presentation. However, the manuscripts dealing with contents beyond the scope of this journal, insufficient matter, no originality, inaccurate data and ideas with basic flaws have to be rejected.

With the committed cooperation of the publisher and the members of the editorial board, the future of JMP appears bright. I look forward to valuable suggestions and scientific contributions from the medical physics community to help us in our joint effort to accelerate this vehicle of communication to climb new heights.
